# Treatment of Hernia*Read before Buffalo Medical Club, May, 1886.

**Published:** 1886-10

**Authors:** William H. Heath

**Affiliations:** Surgeon to Buffalo Hospital of Sisters of Charity


THE BUFFALO MEDICAL AND SURGICAL JOURNAL

Vol. XXVI. OCTOBER, 1886. No. 3

### Original Communications.

### *TREATMENT OF HERNIA.**

By WILLIAM H. HEATH, M. D.

Surgeon to Buffalo Hospital of Sisters of Charity.

Among the anatomical features bearing upon inguinal hernia are to be noted the obliquity of the so-called canal, not only in its direction from above downward and inward, but in its slanting direction, through the abdominal walls, constituting an arrangement not unlike that existing for the entrance of the ureter into the bladder, or the indirect incision occasionally made in evacuating certain fluids. It partakes, therefore, more of the character of a valve than of a canal, which in fact does not exist save as an abnormal condition, and firm uniform outward pressure should in the normal, tend to approximate its anterior and posterior boundaries.

Frederick Treves puts it, "a tract of tissue so arranged as to permit of a body being thrust along it—'a breach,' 'not a doorway.'"

The integrity of this valvular arrangement depends not only on its fascial attachments to the adjacent osseous parts, but

*Read before Buffalo Medical Club, May, 1886.

upon a firm connective tissue which binds the parts mutually together, cements them so to speak, and especially the anterior and posterior walls, and at the deeper part, consequently the formation or protrusion of a hernia is only possible through severing this tissue, which normally has the important function of keeping the two walls in approximation. Under these circumstances the internal ring becomes more pronounced and permits pressure to be exerted against the exposed portion of the anterior wall, while the posterior or internal one is forced inward and away from it at the expense of the tissue in question. Tissue tonicity and strength therefore, is correctly looked upon as having important etiological significance.

The mobility of the posterior as contrasted with the stability of the anterior, and the traction capable of being exerted by the rectus tendon is also to be noted. It permits the margin of the internal ring to become prominent, allows the canal to be effaced in long-standing hernia, and modifies certain forms of treatment.

The frequency of the infirmity creates the strongest interest in its etiology.

The influence of age, sex, occupation, length of the mesentery, and other causes, are fully discussed in surgical text-books. Mr. Wood ingeniously advances the opinion, that in almost all ruptured individuals, there exists a predisposition due to defective formation of the structures of the groin, the consequence of a late descent of the testes, and which is manifested by a loose and bulgy condition of the part, patulous orifices, etc., and that depending on the degree of this defect we have congenital hernia or a predisposition to such an extent, that slight causes are sufficient to effect the injury. This theory is strengthened by the fact that hernia occurs more frequently in men, and in the inguinal region than in women, and that it occurs 33 per cent. more frequently on the same side—the right—that undescended and incarcerated testes occurs. It may be said, however, that hernia are not uncommon in groins that appear in every way normal, and that it gives the idea of a surprising and unusual irregularity in the descent of the testicle, particularly as the delay is generally the consequence of some pathological condition, such as inflammation, adhesion, etc.

Its prevalence among the aged, development among convalescents from exhausting diseases, occurence in those with apparently sound groins, leads to the opinion that it is more than a mere mechanical lesion, and not due to any single factor, but to the result of a combination of conditions, found not only in the abdominal wall, but in the viscera and their attachments, and very considerably to what has been termed tissue tonicity.

The extent of the condition is simply astonishing, and it is not too much to say that, judging from the number of trusses sold, which only represents reducible hernias, and among those who can afford the purchase, the reports of the War Department, text books, statistics furnished by truss societies and other sources, that many millions of men in this country alone, are suffering and are more or less disabled from this defect. Its prevalence also accounts for and affords a rich field for quacks and charlatans, who prey upon the ignorance and sensibilities of this class. In almost every newspaper may be seen the advertisements of traveling hernia doctors, patent trusses guaranteed to cure, and the like.

How thorough then, should be our knowledge and our ability to recognize and treat the malady in its various conditions; yet owing to our miserable system of medical education, I doubt if a large majority of those most likely to be called upon to apply this knowledge, are not sadly wanting, and that many lives through ignorance, are rendered miserable and occasionally lost.

As commonly presented, there should be no difficulty of diagnosis. Blunders are observed, however, every little while, though very often from carelessness, no doubt; within a year I have known hydroceles and varicoceles sent by reputable physicians for trusses, orchitis mistaken for strangulated hernia, and a hydrocele operated on for irreducible herina.

What then, should be our treatment of this condition. I must begin by stating that without doubt the day has passed when the duty of the practitioner, to his patient, terminates with referring him to a druggist to purchase a truss. On the contrary, it can be truthfully stated that it is now in the power of the surgeon to relieve and cure a very large number of those who yet are being consigned by their over-conservative or lethargic medical advisors to wearing a truss, with all its attending discomforts and objections. For reasons well known, up to a comparatively recent time, no operation for the permanent cure of hernia enjoyed, in any sense, the full confidence of the profession, and though no field in surgery has offered more inducements, in none has more work been expended with so little return, operations having been devised again and again only to be abandoned, even by their authors. It is, however, a fact beyond doubt that antiseptic surgery, improved technique, the advance in the treatment of wounds has had its effect in modifying this opinion, and as a consequence, these views are no longer entertained even by some of the most conservative surgeons. Though further statistics are needed to completely establish and justify the proceedings advocated, those that have appeared are certainly of the most encouraging sort.

In view of these facts, it is to be stated that for the average inguinal hernia capable of being securely and comfortably retained by means of a truss, with the exception of the seldom practiced Heatonian method, the application of which, however, is extremely limited, an operation is not as a rule to be advised. To this, however, depending upon peculiar individual conditions, age, occupation, and other considerations, many exceptions are to be taken.

Such of these reducible and retainable hernia, as one of comparatively recent origin, or of reasonable small size, and very particularly where the relative position of the rings to each other are unchanged, the patient being in good health other things being equal, the Hetonian method offers a safe and permanent cure, more satisfactory to the patient as well as surgeon than any other. In forty-nine cases upon which I have operated, I have never witnessed a serious symptom, or anything to cause a regret, and, with certain exceptions, occuring early in my experience, I have every reason to believe in at least 80 per cent. the results were permanent, though my cases have been mainly among the working class. No better evidence is needed, in the absence of the opportunity to examine them in person, than that these very cases have years afterwards referred others to me, while here in Buffalo, a number have since been engaged in daily laborious work without a return of the protrusion.

The traditional prejudice against it and its author, intensified by the offensive style of Dr. Warren's book and claims on the subject, together with its failure to accomplish all claimed for it when tried by the regular profession, the result, however, of its indiscriminate application have altogether combined to relegate it well nigh to oblivion, nevertheless its value in its limited sphere of application is without doubt, and is recognized at home and abroad by many excellent surgeons. Of the operation its merits and objections I have already written, so will not dwell upon it save to remark that it meets the indications and is probably based upon sounder pathology than any other method, viz.: that by creating an adhesive inflammation, proliferation of tissue and contraction, it brings together the anterior and posterior wall of the valve or canal, and occludes the entire passage, without accomplishing which, no operation can be said to be correct in principle.

Reducible hernia, which from the size of their openings, their bulk, or other reason, cannot at all, or without difficulty or with safety and comfort be retained within the abdomen by means of a truss, and particularly in those under middle age, are invariably cases for operative treatment, and such cases are in general prepared to assume some risks. The dangers ever present and perspective in these cases and which cannot be averted are too well known, while the fact that every case of death from strangulation is one from a clearly preventable cause, warrants such risk as attend operative interference.

In these cases many operations have been suggested, the history or description of which would be tedious, and from their complexity difficult to understand.

The best, safest and the one holding out the most inducement from every point, is that now known as the direct method of dissection, and which consists in exposing the external ring and sac, which is isolated, ligatured at its neck, and occasionally removed, following which the pillows of the ring are sutured with wire or catgut, after freshening their edges, or no, as the judgment of the operator may incline. As practiced to-day it is not exactly the operation of any one man, but has been advocated by many, though to the senior Gross is probably due the honor of suggesting and operating. It is variously modified by surgeons in leaving or excising the sac, either in part or entirely, or ligating in one or several places, according to taste or the peculiarities of the case. The most difficult feature consists in dissecting out and isolating the sac, especially from the coverings of the cord from which it is at times inseparable, and which is more easily described than accomplished. Closure of the external abdominal ring is the bulwark upon which security depends, and though unphilosophical and when any canal remains inferior to Wood's suture which closes the entire passage it is practically satisfactory, and should furthermore be performed in every case of herniotomy for strangulated hernia.

The class of hernia just defined when left untreated sooner or later become irreducible, by which is understood that in consequence of increased bulk, contraction of the neck of the sac, or adhesions within or without it, it cannot be returned to the cavity of the abdomen. This state at one time of its existence preventable, is always one of danger from strangulation or injury. From their tendency to increase steadily in size, enormous cases are occasionally met with, one of the most notable occurring in the person of the distinguished historian Gibbon, whose pylorus rested at the mouth of the sac. Operative treatment, with a view of rendering it either reducible or obtaining a permanent cure, is, in the majority of instances, unjustifiable, on account of the difficulties and hindrance to success and the danger to life, conditions which are always present in varying degrees.

Retention by means of an abdominal supporter in corpulent persons, to diminish pressure from above, a suspensory, to retain the protrusion from dragging and preferably of some hard material, is the best line of treatment, to which may be added a quiet careful life, avoiding intestinal derangement and anything like sudden activity. I am aware that some surgeons think otherwise, and take, what may be called, desperate chances. In the light of my own experience and observation, however, I am not prepared to take these chances, and consider it much the best to palliate the evil, particularly in adults.

All hernia, sooner or latter, become strangulated, the average duration of inguinal being about twenty years. This strangulation occurs under two different forms, differing in mechanism, symptoms and treatment. In the one acute strangulation, it occurs immediately upon descent; perhaps the very first, its essential characteristics being violent symptoms, sudden onset, occurring in an enterocele, and, as a result of sudden arterial occlusion, mortification of gut.

The other, on the contrary, occurs generally in old irreducible hernia, containing omentum, as well as intestine, its essential characteristic, in comparsion, being slow and chronic and due variously to alterations in size of tumor, adhesions, and but rarely to the descent of additional viscera—the alterations in the tumor being more the consequence of various obstructions than arterial occlusion. One or two points in the mechanism of acute strangulation seem not to be generally understood.

Whether it be a hernia, strangulated in its formation or occuring to an already existing one, essentially it is the same, the conditions present being a tumor, with obstruction of its circulation and compression of its nerves, at a point designated as the neck generally, and the result of sudden increase in its size. The conclusion generally inferred is that this neck is much the active agent in bringing this about, whereas nothing of the kind is the case, it is in fact, a passive agent, incapable of strangulating anything, although it has been said muscular fibres are occassionally found there.

This sudden increase in size at its narrowest part, causing a gripping or pinching as it were, and which is the whole difficulty, is mainly due to the scarcely heard mentioned mesentery, which belongs to the knuckle of gut forced down. This should be clearly understood, it is not a new coil which gets down into the sac in addition to the one already down, thus making two coils, but is more of the same coil. In the case of an existing hernia redescending, the original coil and mesentery were not sufficiently bulky to cause any difficulty when down, but the additional section brings with it a further and proportionately larger instalment of mesentery, which is crowded or pulled in where there was barely room for what was present before; it is also to the interference of the circulation, through mesentery that congestion and strangulation is due, rather than to compression of gut itself.

The general line of treatment in this condition is, in the main, clearly established; though in practice is often deviated from.

The first interference is in the form of taxis, concerning which it may be said that notwithstanding all written and cautioned concerning it, is to-day made too roughly, too prolonged, and often ignorantly, and that it contributes to the fatality of herniotomy more than any other cause. That it is over-done there can be no doubt, the temptation to do more or try again being very considerable. Of adjunct means and absurd therapeutics, peculiar positions, local applications, etc., which have for their object contraction by cold or relaxation by heat, little need be said, their value, depending entirely on the character of the strangulation. Aspiration, however, the most valuable of all our means to reduce the tension and volume of gas, is hardly ever used. By it fluid as well as gas can be withdrawn, and is decidedly safer than prolonged taxis, and I have used it a number of times, without disappointment.

As a matter of fact it is interesting to note that old hernia are often capricious and return of their own volition after the most willful attempts, also that patients, the subjects of hernia, are frequently adepts in returning their own protrusions, and should be encouraged to make moderate trial to relieve themselves.

As much of the mortality of herniotomy can be attributed to delay, and as there are no symptoms by which the condition of the hernial contents can be positively known, and therefore, how late operative measures can be deferred, it should not be left as a last resort but should be resorted to early, certainly before tenderness is added to tympanites. Properly, performed it is not dangerous, in fact, if the sac be not opened it becomes merely a modified taxis, nor should it be omitted in the most desperate cases, recoveries occasionally taking place under the most unfavorable conditions, in fact so long as the patient is not moribund it should be practiced.

So long as there are cases which only admit of palliative treatment, and so long as we can bring about a cure in a large number of ruptured children by the use of trusses, so long will their construction and application be a matter of importance and it is to be regretted that their selection and fitting, a matter not in any way undignified or unprofessional, should have been allowed to pass from the hands of the surgeon into the domain of druggists and others who only rarely have a correct knowledge of the infirmity, no professional reputation to sustain, and whose only object is generally the sale of the appliance. In consequence of this some hernia are injured, others made infinitely worse, and very many unnecessarily uncomfortable. Without going into the matter at all, in a general way, it may be stated that a truss should combine efficiency and safety with comfort; efficiency in retaining the protrusion within the abdomen and prevent its escape under all ordinary movements; safety in accomplishing this without injury to the parts; with comfort that it fit like a garment and produce no discomfort. These points are best obtained by a suitable spring and pad, which is the most important part and which should be oval, to allow a better adaptation and press over the entire canal, convex to a moderate degree and of hard wood, to better maintain its shape and position. Soft pads are objectionable in that they absorb moisture change form and are dirty, ivory ones in being too slippery and expensive, hard rubber in their likelihood to chafe. Of the many varieties found in the shops those that are double and fasten behind are in general, superior in the points named. The well known "Lyman" truss, made after designs and suggestions of my own, will be found to fulfil the indications very satisfactorily.

As is well known the application of trusses in the adult is merely palliative, though occasionally, when worn at once in sudden hernia from injury, a cure is effected. With children of reasonable age it is different, they generally effecting a cure by obliteration, adhesion, and by giving support to the structures, during the period of development. Hernia in children under one year or before they can crawl and walk, in my judgment, are best without trusses. I am satisfied more harm is done from the irritation of trusses than any benefit, and could quote many cases from my note book in support of this opinion. In these cases the protrusion can be restrained if not retained by a loose skein of worsted or flannel bandage and even if not retained no great harm is done.

Fat men with large abdomens are the most difficult to fit either comfortably or securely, neither are they good cases for operation on general principles. A truss which would also combine in itself the properties of an abdominal supporter would meet a decided want.

Among the injurious consequences of body-fitting trusses may be mentioned: Atrophy from too great pressure, spreading open the rings from too great convexity of pad, pain and chafing, atrophy, neuralgia of testicle, etc., from pressure on cord and liability to sudden descent and strangulation, through a false sense of security.

Inasmuch as it is the present custom among many to leave this important though apparently simple duty to the trade, the best precaution is to designate a thoroughly reputable concern to the patient, and after being fitted have him return for examination as it is common for many alterations to be necessary, and they are most evident to the medical man.

